# Necrotizing Pancreatitis With Concurrent Clostridioides difficile Infection

**DOI:** 10.7759/cureus.115249

**Published:** 2026-08-26

**Authors:** Maison D'Amelio, Kavya Avancha, Joseph Hassler, Madhumita Baskaran, Wilfredo Grana

**Affiliations:** 1 Research, Alabama College of Osteopathic Medicine, Dothan, USA; 2 Medicine, Alabama College of Osteopathic Medicine, Dothan, USA; 3 Internal Medicine, Regional Medical Center, Anniston, USA

**Keywords:** acute necrotizing pancreatitis, c diff infection, delayed cholecystectomy, gallstone-induced necrotizing pancreatitis, post cholecystectomy complications

## Abstract

Necrotizing pancreatitis is a severe complication of acute pancreatitis that may not become radiographically evident until several days after symptom onset. Although progression from gallstone pancreatitis is well recognized, concurrent postoperative complications and *Clostridioides difficile* infection can obscure the clinical picture and delay diagnosis. This case adds to the literature by demonstrating how overlapping postoperative findings and concurrent infection masked the evolution of necrotizing pancreatitis despite initial clinical improvement and illustrating treatment approaches in such situations.

A 61-year-old man presented with worsening right upper quadrant abdominal pain radiating to the back and was diagnosed with gallstone pancreatitis secondary to choledocholithiasis. He underwent endoscopic retrograde cholangiopancreatography (ERCP) with sphincterotomy and stone extraction followed by delayed laparoscopic cholecystectomy. After initial improvement and discharge, he returned with severe abdominal pain, nausea, vomiting, and systemic inflammatory findings. Exploratory laparotomy revealed chemical peritonitis of pancreatic origin without biliary leak or enteric perforation. During hospitalization, he developed *Clostridioides difficile* colitis, further complicating interpretation of persistent abdominal pain, leukocytosis, and inflammatory markers. Interval MRI ultimately demonstrated sterile peripancreatic necrosis and fluid collections consistent with necrotizing pancreatitis. The patient was managed with bowel rest, total parenteral nutrition, drainage of peripancreatic fluid, treatment of *Clostridioides difficile* infection, serial imaging, and multidisciplinary care involving surgery, gastroenterology, infectious disease, critical care, and internal medicine. His symptoms gradually improved, and he was discharged with outpatient surgical follow-up.

This case highlights the unpredictable progression of gallstone pancreatitis to necrotizing pancreatitis despite early intervention and apparent clinical recovery. Persistent or worsening postoperative abdominal pain should prompt continued reassessment, as concurrent infections such as *Clostridioides difficile* may obscure an evolving pancreatic process. Serial laboratory evaluation, interval imaging, and multidisciplinary collaboration are essential for timely diagnosis and improved patient outcomes in complex cases.

## Introduction

Acute pancreatitis is a sudden inflammation of the pancreas. It is a common gastrointestinal disorder that has several different causes, with gallstones and alcohol consumption the two most likely [1]. Other less common causes of acute pancreatitis are certain medications, hypertriglyceridemia, hypercalcemia, pancreatic cancer, and injuries from endoscopy, trauma, or surgery [1]. Gallstones constitute up to 40% of acute pancreatitis cases in the Western world and typically present with a mild, self-limiting clinical course [2]. However, some patients may develop severe forms of acute pancreatitis. This can be assessed using the Bedside Index of Severity in Acute Pancreatitis (BISAP) Score [3] with one point for each of the following: blood urea nitrogen (BUN) >25 mg/dL, impaired mental status which corresponds to <15 on the Glasgow Coma Scale, systemic inflammatory response syndrome (SIRS) present, age over 60 years, and pleural effusion present on imaging. A BISAP score of greater than 3 indicates a higher risk of mortality and increased risk for severe organ damage.

Necrotizing pancreatitis is a severe form of acute pancreatitis, in which sections of the pancreas undergo necrosis due to inadequate blood flow [4]. Approximately 20% of acute pancreatitis cases progress into necrotizing pancreatitis. This disease has significant morbidity and mortality, with survival rates depending on the level of necrosis of the pancreas [4]. Progressing from acute pancreatitis to necrotizing pancreatitis can be unpredictable. An initial presentation of acute pancreatitis does not always lead to such disease severity, as the necrosis of the pancreas may evolve after several days [5]. Furthermore, concurrent complications, such as *Clostridioides difficile* infection, and overlapping postoperative findings, including abdominal pain and leukocytosis, can blur the clinical picture and delay recognition of evolving pancreatic necrosis. For example, some patients have abdominal pain after endoscopic retrograde cholangiopancreatography (ERCP) due to perforation or discomfort from air insufflation. Patients with perforation may present with bloating, abdominal pain, fever, tachycardia, and leukocytosis [6]. Several of these symptoms are similar to the symptoms of pancreatitis. Similarly, *Clostridioides difficile* infection can present with abdominal pain, fever, abdominal distension, ileus, and leukocytosis, which may overlap with the clinical manifestations of pancreatitis [7]. These overlapping symptoms can therefore make it challenging to distinguish expected postoperative complications or concurrent complications from progression of pancreatic disease.

This case report presents a 61-year-old male who had a delayed diagnosis of necrotizing pancreatitis after an initial diagnosis of gallstone-related acute pancreatitis. Throughout his multiple emergency room visits and hospitalizations, he received an ERCP, underwent a cholecystectomy, had complications of postoperative abdominal pain after initial improvement, and developed a Clostridioides difficile infection. The patient’s disease course progressed in an unexpected and complicated manner that obscured the diagnosis of necrotizing pancreatitis. This case highlights the diagnostic challenge of recognizing evolving pancreatic necrosis when clinical findings overlap with postoperative and concurrent complications. The case report also emphasizes the importance of continued clinical vigilance when symptoms persist or recur despite initial clinical improvement, as well as the value of multidisciplinary management in complex cases. 

## Case presentation

The patient is a 61-year-old Caucasian male with a past medical history of gastroesophageal reflux disease (GERD), hypertension, and hyperlipidemia who originally presented to the emergency room for abdominal pain after breakfast and a dose of tirzepatide. He complained of right upper quadrant pain and 7/10 chest pain. Electrocardiogram (ECG) showed normal sinus rhythm on initial presentation, and a chest X-ray showed no acute chest findings. Lab values on presentation included elevated bilirubin 2.0 mg/dL (0.2-1.0), aspartate aminotransferase (AST) 134 unit/L (8-33), and alanine transaminase (ALT) 78 unit/L (10-49). One 80 milligrams (mg) dose of simethicone was given, and the patient was sent home with a diagnosis of gas pains and a prescription for simethicone.

**Figure 1:**
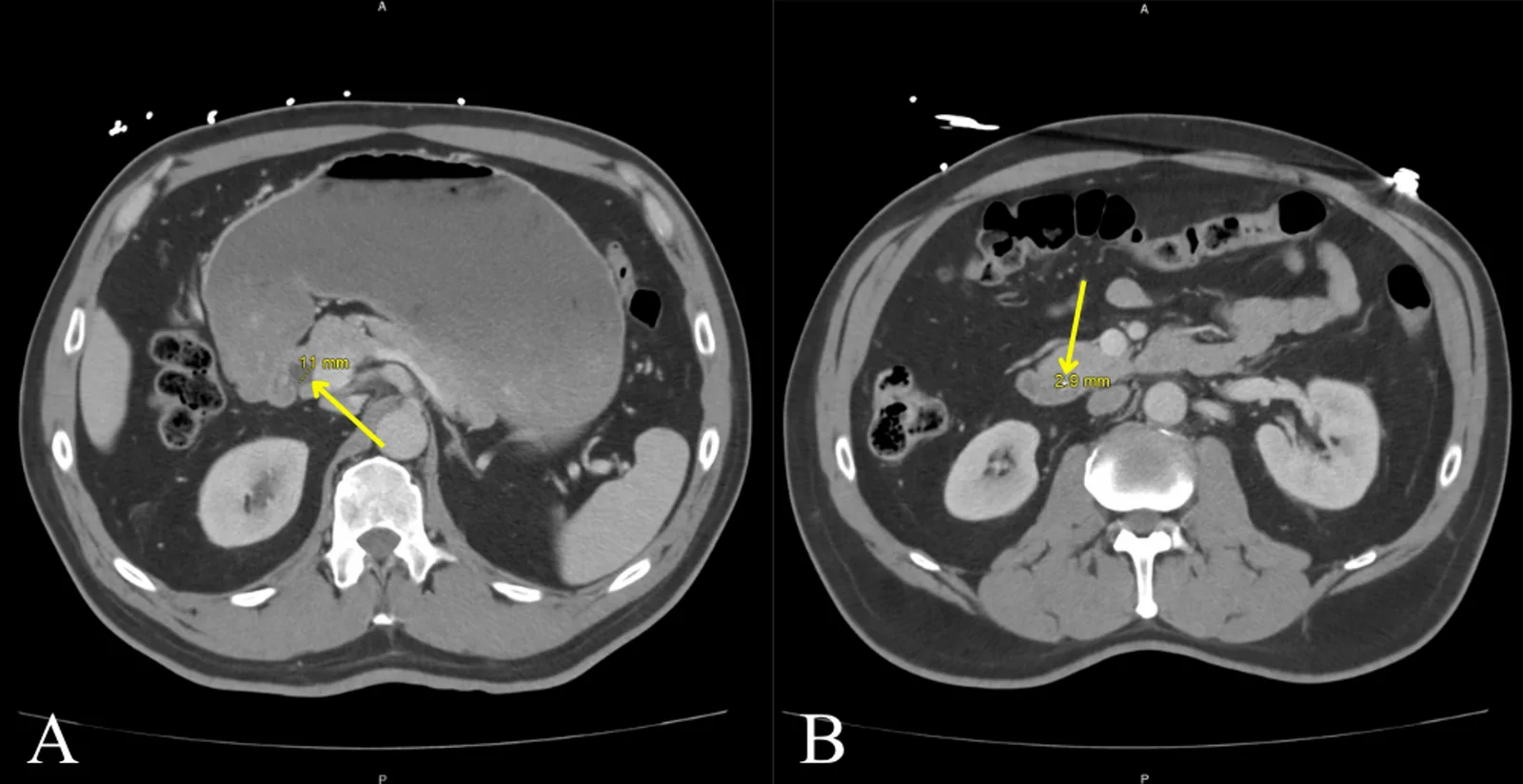
Axial contrast‑enhanced CT of the abdomen demonstrating choledocholithiasis and mild intrahepatic bile duct dilatation (Panel A) The arrow marks a calcified stone at the distal common bile duct near the ampulla, measuring approximately 11 mm. (Panel B) The arrow indicates a more proximal common bile duct stone measuring approximately 2.9 mm, with associated mild prominence of the extrahepatic bile duct, consistent with the reported choledocholithiasis.

He returned the next day when the abdominal pain got worse and now described it as a sharp pain to the right side of his abdomen radiating to his back. Again, an ECG and chest X-ray were negative, but a computed tomography (CT) of the abdomen found a distended gallbladder with gallstones, a 2.9mm ampullary choledocholith, and a common bile duct dilation of 11mm (Figure 1). His labs supported these findings, with elevated lipase, bilirubin, alkaline phosphatase, and liver enzymes (Table 1). An esophagogastroduodenoscopy (EGD), endoscopic ultrasound (EUS), and ERCP were performed on Day 0. Gastritis was noted in the gastric body and antrum. Multiple gallstones were noted, causing a common bile duct dilation of 12 mm, with a subsequent sphincterotomy with removal of the calculus performed. He developed aspiration pneumonia in the right lower lobe following this procedure, so the laparoscopic cholecystectomy with omental biopsy was scheduled for Day 4 rather than the next day. He was also started on amoxicillin/clavulanic acid, piperacillin/tazobactam, and given one dose of cefazolin. His labs were assessed every day (Table 1). He was discharged the following day with no acute complaints on amoxicillin/clavulanic acid.

**Table 1:** Key laboratory trends during the initial hospitalization Selected laboratory values from days 0–5 of the first admission. Marked elevations in lipase, aminotransferases (AST, ALT), alkaline phosphatase, and total bilirubin on days 0-2 support the diagnosis of acute gallstone pancreatitis with cholestasis. Persistent leukocytosis during this period reflects systemic inflammation. Mild hypocalcemia and transient hypokalemia are also present and gradually improve over the first several hospital days. Abnormal values are shown in bold. K: potassium; Ca: calcium; AST: aspartate aminotransferase; ALT: alanine aminotransferase; ALP: alkaline phosphatase

	Reference	Day 0	Day 1	Day 2	Day 3	Day 4	Day 5
K	3.5-5.1 mmo/L	3.2	3.9	3.7	3.5	3.8	3.8
Ca	8.7-10.4 mg/dL	9.6	8.6	8.4	8.5	8.3	8.6
Lipase	12-53 units/L	57	>3500	1480	125	45	38
AST	8-33 unit/L	454	335	115	43	26	42
ALT	10-49 unit/L	348	462	297	138	77	68
ALP	46-116 unit/L	125	169	151	96	68	70
Bilirubin	0.2-1.0 mg/dL	4.3	4.7	3.3	3	2.1	1.1
WBC	4.5-10.4 10^3^/μL	8.2	18.4	18.8	14	11.8	13.3

Two nights after his discharge, the patient felt a “tear and like someone shot fire across from my right side to left side like I’d been sprayed” when he got up to use the restroom. This was accompanied by feeling hot, sweating profusely to his back, nausea, and vomiting, further prompting his home health nurse to advise him to present to the emergency room again. The physical exam was unremarkable besides moderate right lower quadrant tenderness and diminished bowel sounds. On readmission, arterial blood gas analysis demonstrated a mildly alkalemic primary respiratory alkalosis with hypoxemia (pH 7.47, partial pressure of carbon dioxide (PaCO₂) 29.0 mmHg, partial pressure of oxygen (PaO₂) 62.9 mmHg). Serum studies showed a creatinine of 1.76 mg/dL (reference 0.55-1.30), total bilirubin 1.3 mg/dL (0.2-1.0), lipase 155 U/L (12-53), amylase 291 U/L (30-118), and lactic acid 2.4 mmol/L (0.4-2.0). These findings were consistent with recurrent acute pancreatitis and early organ dysfunction. The BISAP score on admission was 2, based on the presence of systemic inflammatory response syndrome and age > 60 years, indicating an increased risk of severe disease.

**Figure 2:**
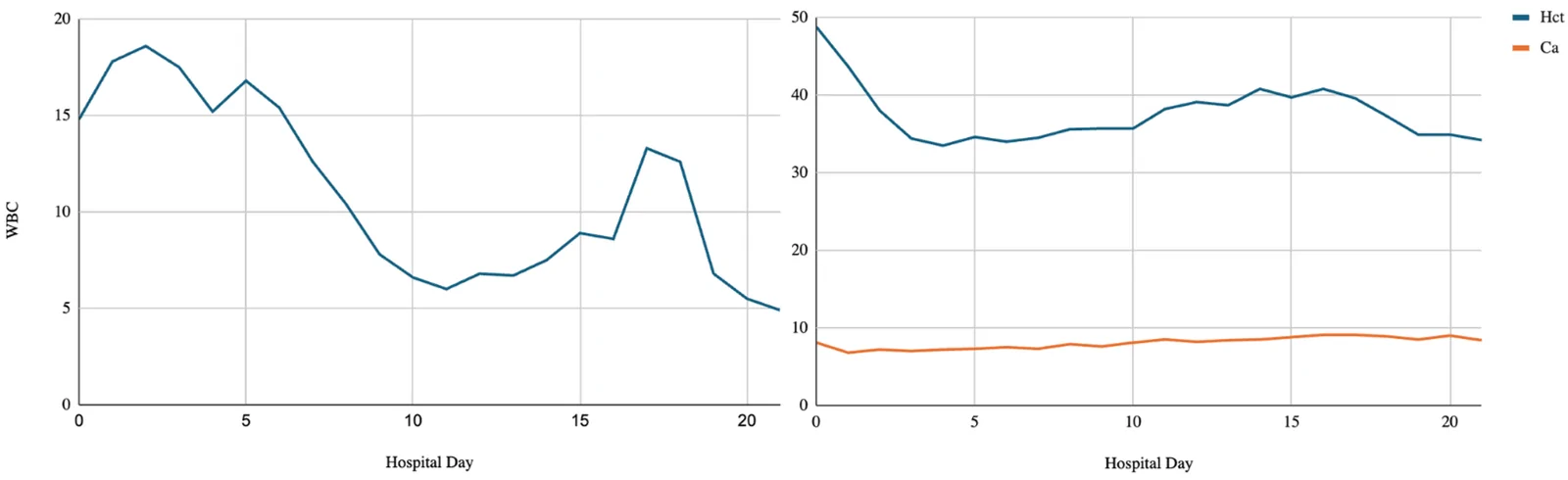
Trends in systemic inflammation and severity markers during hospitalization Daily laboratory trends in a patient with necrotizing gallstone pancreatitis and concurrent *Clostridioides difficile *infection. Left panel: White blood cell (WBC) count by hospital day demonstrates marked leukocytosis at presentation with gradual improvement, followed by a secondary rise around days 17–18 corresponding to recurrent infectious/inflammatory activity, and subsequent normalization. Right panel: Hematocrit (Hct, blue line) and total serum calcium (Ca, orange line) by hospital day. Hct is initially elevated, then falls and remains in the low‑normal range, reflecting early hemoconcentration with subsequent anemia of critical illness and hemodilution. Calcium is depressed from admission and remains below the reference range until approximately day 15, consistent with severe necrotizing pancreatitis, before normalizing during recovery.

Labs were trended and are displayed in Table 2 and Figure 2. On admission (day 0), serum sodium, potassium, chloride, magnesium, phosphate, total protein, and albumin were within or slightly below the reference ranges. The only liver test abnormality was a mildly elevated total bilirubin of 1.3 mg/dL, with normal aspartate aminotransferase (AST), alanine aminotransferase (ALT), and alkaline phosphatase (ALP), consistent with transient biliary obstruction. Lactate was modestly elevated at 2.4 mmol/L and normalized thereafter. Creatinine was 1.76 mg/dL on arrival but rapidly improved and remained within the reference range for the remainder of the hospitalization, and blood urea nitrogen stayed normal or low, indicating preserved renal function. Electrolyte disturbances during the hospital course were mild and transient. Potassium was briefly low on days 0 and 4 (nadir 3.1 mmol/L) and corrected with replacement. Sodium values were generally within the reference range, with only mild late hypernatremia (maximum 145-146 mmol/L) during the recovery phase, attributed to free‑water deficit and resolved with fluid adjustment. Albumin and total protein were persistently low in the first half of the admission, reflecting systemic inflammation and catabolism, and gradually normalized as the patient improved. Platelet counts were elevated early, peaking at 574 ×10⁹/L, then trended downward but remained within or near the upper reference range, consistent with a reactive thrombocytosis.

**Table 2:** Trends in pancreatic enzymes and C‑reactive protein during hospitalization Serum pancreatic enzyme and inflammatory marker values over the hospital course. Lipase and amylase were markedly elevated on admission (day 0) and remained above the reference range on day 3, consistent with acute gallstone pancreatitis. Lipase peaked on day 6 and subsequently normalized, while amylase returned to the reference range after day 3 and was not re‑measured. C‑reactive protein (CRP) was first obtained on day 6, when it was markedly elevated, and declined steadily on days 8, 9, and 13 in parallel with clinical improvement and stabilization of necrotizing pancreatitis and *Clostridioides difficile *infection.

Hospital day	Lipase (U/L)	Amylase (U/L)	CRP (mg/L)
0	155	291	–
3	171	179	–
6	346	–	137.2
8	–	–	100.51
9	–	–	82.24
13	–	–	38.6

**Figure 3:**
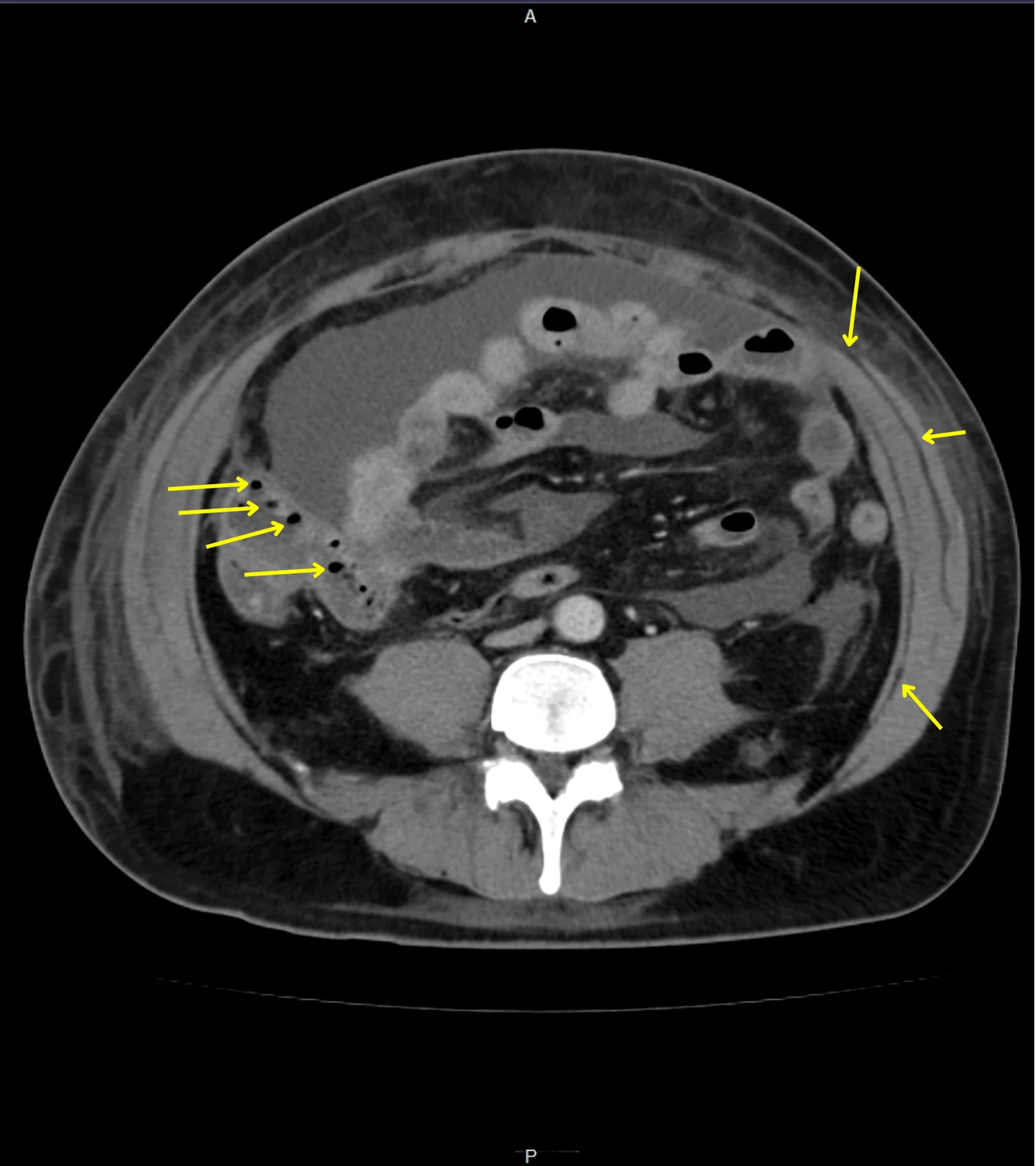
Axial contrast‑enhanced CT of the abdomen demonstrating diffuse intraperitoneal free fluid Arrows along the right abdominal wall highlight low‑attenuation fluid layering in the left paracolic gutter and anterior abdomen, while the left cluster of arrows indicates additional dependent free fluid outlining small‑bowel loops, consistent with the reported intraperitoneal and mesenteric fluid collections following recent cholecystectomy.

On Day 0 of his second admission, the patient had a CT scan which showed diffuse intraperitoneal free fluid and pneumobilia (Figure 3). This was followed by a diagnostic laparoscopy converted to an exploratory laparotomy. Drainage of peripancreatic fluid collection and abdominal washout were performed with no findings of biliary leak, enteric perforation, or abscess. Chemical peritonitis likely from pancreatic origin was noted, and a firm and indurated pancreas warranted placement of a Jackson-Pratt (JP) drain. He was then admitted to the intensive care unit (ICU) with a nasogastric tube and on total parenteral nutrition. He began vancomycin and piperacillin/tazobactam, and was given one dose of cefazolin. A culture of the body fluid sampled during the laparoscopy was obtained on Day 1 and was negative. The patient developed diarrhea and, due to his recent antibiotic use, was tested for *C. difficile *toxin. Microbiology on Day 3 was positive for *C. difficile *toxin, and the patient was started on intravenous (IV) metronidazole since he was on nothing-by-mouth orders. He was confused following surgery and accidentally pulled out his nasogastric tube on Day 4. Haloperidol was given that night to assist with sleep since the patient had not been able to sleep much since surgery. On Day 5, the patient was more alert and less confused. A microbiology report of CSF obtained on Day 5 had rare *Candida krusei *isolated, so he was started on micafungin. The following day, the patient’s confusion was resolved. His bowel movements were no longer watery, but he denied any abdominal pain. 

**Figure 4:**
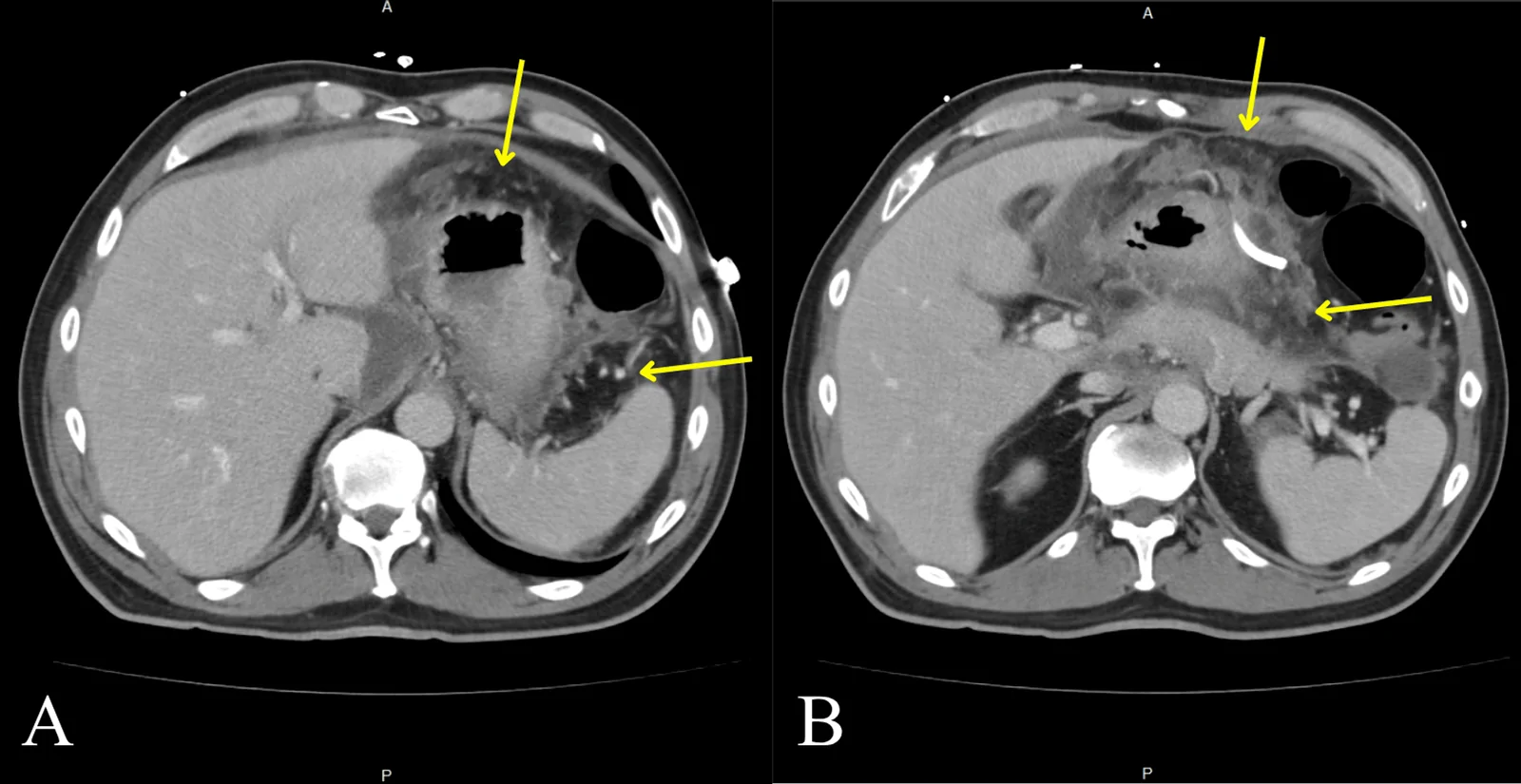
Axial contrast-enhanced CT of the upper abdomen demonstrating appearances consistent with sequelae of pancreatitis with sterile peripancreatic necrosis Axial contrast‑enhanced CT of the upper abdomen (panel A) demonstrating heterogeneous low‑attenuation peripancreatic collections (upper arrow) in the lesser sac posterior to the stomach, with extension of fluid and necrotic tissue along the medial aspect of the spleen (lower arrow), consistent with sterile peripancreatic necrosis and organized fluid collections in the setting of pancreatitis. Axial contrast‑enhanced CT of the upper abdomen (panel B) demonstrating heterogeneous low‑attenuation peripancreatic collections replacing the expected pancreatic contour. The superior arrow indicates necrotic fluid and soft tissue extending anteriorly from the pancreatic bed, while the inferior arrow highlights a more focal peripancreatic collection posterior to the stomach, consistent with organized sterile peripancreatic necrosis in pancreatitis.

**Figure 5:**
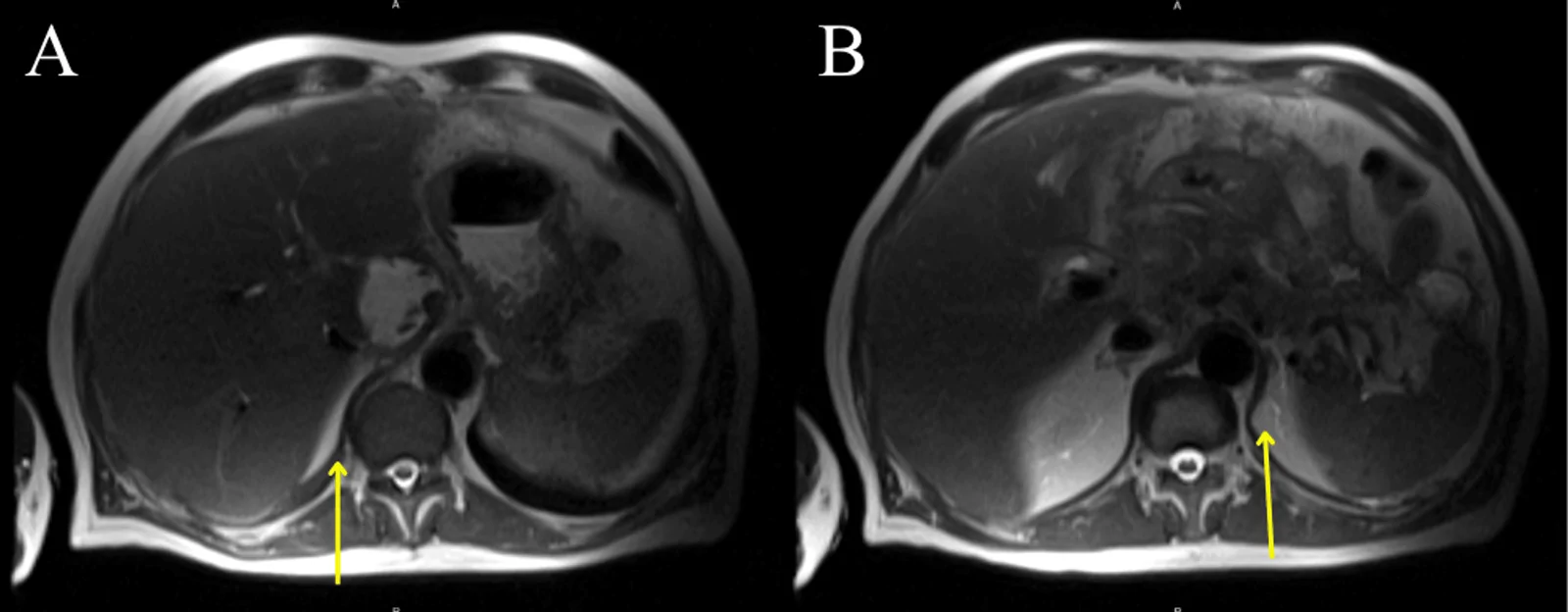
Axial MR images of the upper abdomen without contrast (Panel A) Fluid‑signal collection in the upper abdomen anterior to the vertebral body (arrow), compatible with a small abdominal pseudocyst. (Panel B) Peripancreatic fluid‑signal collection adjacent to the pancreatic tail and splenic hilum (arrow), consistent with a peripancreatic pseudocyst in the setting of pancreatitis.

He was moved to the Progressive Care Unit and was able to get up and move around with assistance within his room due to *C. difficile *precautions; his abdomen was still somewhat distended, but not painful. As he was coming off of nothing-by-mouth orders, he started to have PO vancomycin rather than IV. He said that he had not been sleeping well on Day 7 with intermittent diarrhea, so ziprasidone was ordered. The nasogastric tube was clamped and subsequently removed on Day 11 and Day 12. He was advancing in diet with little to no abdominal pain, but developed a low-grade temperature accompanied by nausea and vomiting on Day 15. On Day 16, a CT indicated appearances consistent with sequelae of pancreatitis with sterile peripancreatic necrosis in the lesser sac and adjacent to the spleen with sterile peripancreatic fluid collections in the upper abdomen (Figure 4). The patient was placed on nothing by mouth orders and then was re-advanced over the next few days. MRI (magnetic resonance imaging) of the abdomen on Day 21 found mild peripancreatic inflammatory changes with a small upper abdominal fluid collection compatible with a pseudocyst (Figure 5). The patient was cleared for discharge and was instructed to follow up with surgery for removal of the J tube and the wound vacuum-assisted closure (VAC).

## Discussion

The patient presented to the emergency room with gallstone-related pancreatitis, which led to an ERCP being performed and a delayed cholecystectomy. Initially, there was improvement, but his severe abdominal pain returned, prompting an exploratory laparotomy. His subsequent course was complicated by *Clostridioides difficile *infection, which introduced additional abdominal and systemic inflammatory findings that overlapped with those of evolving pancreatic disease. These concurrent postoperative and infectious processes complicated clinical assessment and may have contributed to delayed recognition of necrotizing pancreatitis.

Gallstones are one of the most common causes of pancreatitis [8]. Pancreatitis occurs due to a gallstone obstructing the Ampulla of Vater. This blocks pancreatic duct outflow and increases the intraductal pressure, leading to ductal hypertension. Pancreatic secretions and bile reflux back into the pancreas, causing premature enzyme activation. The premature enzymes result in pancreatic inflammation and autodigestion. The progression of acute pancreatitis into necrotizing pancreatitis occurs due to the amplification of inflammatory-driven tissue injury, leading to damage of pancreatic microvasculature. The destruction of pancreatic microvasculature causes hypoperfusion of the pancreas, leading to necrosis, which can progress in 15-20% of cases of gallstone pancreatitis [5]. Risk factors of necrotizing pancreatitis are obesity, age over 55 years, SIRS, early organ failure, hemoconcentration, elevated inflammatory markers, and comorbidities such as diabetes mellitus, hypertriglyceridemia, and cardiovascular disease. In addition, inflammation after an ERCP and surgery can exacerbate the progression to necrotizing pancreatitis.

The diagnostic challenges were due to the pancreatitis being necrotizing in nature, complicated by a concurrent *C. difficile *infection. Early imaging in pancreatitis might not detect local complications as well as CT completed 5 or more days after onset [9]. Initial unremarkable imaging made it difficult to detect pancreatitis in this patient. The pancreatitis was detected on exploratory laparotomy, but not on initial imaging, and also had an overriding *C. difficile *infection. This concurrent inflammatory infection worsened the abdominal pain and leukocytosis.

Management challenges began with the delay of the cholecystectomy due to aspiration pneumonia following the ERCP. Typical bedside approach to acute pancreatitis includes calculating a BISAP score and aggressive fluid resuscitation on admission, serial reassessments including assessing hematocrit and urine output. For high-risk patients, providers should consider ICU placement, ERCP as needed to remove gallstones, and supportive care emphasis. In patients with gallstone pancreatitis, cholecystectomy within the early window of 24-48 hours when compared to a late cholecystectomy of 4-6 weeks after onset leads to a shorter hospital stay and decreased risk of biliary complications, but did not find a significant difference in intraoperative or postoperative complications [10]. The evolution of pancreatitis to necrotic pancreatitis may have been due to the increased risk of biliary complications following a delayed cholecystectomy or simply a post-cholecystectomy complication. However, the specific factors contributing to progression to necrotizing pancreatitis in this patient remain uncertain. Once the exploratory laparotomy following the pancreatitis flare-up concluded, the *C. difficile *infection, which was most likely due to the longer course of antibiotics leading up to and after the cholecystectomy, began to complicate the case. The treatment team aimed to continue to treat with antibiotics with a balance between infection control and risk of worsening the *C. difficile *infection due to the patient’s nothing-by-mouth status and bowel rest. The treatment team utilized a multidisciplinary approach to this case involving Gastroenterology, Surgery, Infectious Disease, and Critical Care all collaborating with the Internal Medicine doctor.

The challenges from this unexpected and complicated case elucidate certain clinical points and insights that can improve the outcomes of future patients. One main point to consider is that acute pancreatitis can progress to necrotizing pancreatitis after initial clinical improvement due to the fact that while necrosis evolves, it may not be evident for 48-72 hours. Although this is a known complication, the progression of disease in this patient was complex, making it an instructive case. The physicians on the case were anticipating discharging the patient soon but were halted due to an MRI that indicated a necrotic pancreas. This displays that acute pancreatitis is unpredictable and can progress in various ways, which is important for clinicians to consider. Furthermore, early imaging often does not exhibit pancreatic necrosis. Usually, the necrosis becomes more evident after 72 hours from symptom onset [11].

Additionally, it is important to emphasize that postoperative pain and concurrent infections can mask evolving pancreatitis. The patient experienced “searing” abdominal pain after his cholecystectomy, causing him to return to the hospital to receive a subsequent exploratory laparotomy and then develop *C. difficile *during his hospital stay. These incidents presented a diagnostic challenge as they obscured the diagnosis of necrotizing pancreatitis, as the diseases had overlapping symptoms. It becomes difficult to interpret abdominal pain, systemic markers, and leukocytosis as there are many disease processes occurring. This makes it hard to decipher what disease process is causing which symptoms. It is important to reassess the progress of pancreatitis and maintain vigilant monitoring as the disease can continue to evolve, which includes repeated lab work, interval imaging, and evaluation. In addition, coordinated efforts within multidisciplinary teams such as General Surgery, Gastroenterology, Internal Medicine, and Infectious Disease can help monitor the patient better and quickly identify progressing disease processes. 

Similar case reports depict the unpredictable clinical course of pancreatitis after stabilization of the patient. A 2025 case report discusses a patient who developed post-cholecystectomy pancreatitis after an open cholecystectomy. After a period of stabilization, the patient unexpectedly developed multi-organ failure and eventually a fatal cardiopulmonary arrest [12]. Another case report describes a patient who developed necrotizing pancreatitis after a laparoscopic cholecystectomy. The patient, after a week of hospitalization, had clinically improved but then returned to the hospital with sepsis two weeks later [13]. These case reports demonstrate that the evolution of pancreatitis can be unpredictable, and it is beneficial for clinicians to understand that the disease may progress rapidly after a deceptive stabilization of the patient. However, the limited number of case reports prevents conclusions regarding the frequency or predictors of this clinical course. Our case adds to the literature by exhibiting how postoperative findings and concurrent *C. difficile *infection may further complicate recognition of evolving pancreatic necrosis. 

This case study has limitations due to it being only one case, but the featured patient did not have any comorbidities, which can be used to study the evolution of pancreatitis in similar patient populations. The specific antibiotics or medications given during the first admission with the ERCP, aspiration pneumonia, and delayed cholecystectomy might have confounded the final diagnosis of necrotic pancreatitis and concurrent *C. difficile*. 

## Conclusions

This case report depicts the evolving nature of acute pancreatitis and how necrotizing pancreatitis can occur despite initial clinical improvement. In addition, this case presents diagnostic challenges as the patient’s postoperative severe abdominal pain and concurrent Clostridioides difficile infection obscured the detection of progression of the acute pancreatitis to necrotizing pancreatitis. The hospital course of this patient highlights the importance of considering necrotizing pancreatitis in the differential diagnosis, particularly with worsening symptoms and insignificant imaging findings. This case report also illustrates that it may be useful to repeat laboratory testing, obtain interval imaging, reevaluate the patient, and collaborate with multidisciplinary teams to identify and manage the developing disease process. The ability to diagnose the progression of pancreatitis in complex settings may help guide timely management. However, as a single case report, these conclusions may not be generalizable to all patients with acute pancreatitis. Further studies are needed to better understand the clinical factors associated with delayed recognition of necrotizing pancreatitis and to determine strategies that may improve outcomes.
